# Supplementary material for: Assessment of in vivo bone microarchitecture changes in an anti-TNFα treated psoriatic arthritic patient
Source: PLoS One. 2021 May 19;16(5):e0251788. doi: 10.1371/journal.pone.0251788 (PMC8133422; doi:10.1371/journal.pone.0251788)
Supplement: Attachment — Submitted filename: response to Reviewers.docx [file pone.0251788.s001.docx]

**Detailed answers to Reviewers**

**Detailed answers to *Reviewer 1***

This is a very interesting work. In my opinion it highlights a result that could have enormous application value in clinical practice. Therefore some "inaccuracies" need to be corrected:

In the paragraph Introduction, the Authors introduce the subject making some statements, some of which in my opinion, need to be reviewed.

- **Point 1.** First of all we know that sites highlighted by ultrasound may be clinically non-active. So this should be specified in the text : The most commonly involved sites by ultrasound assessment are………………(5).

***We have now specified that ultrasound can detect the most common PsA sites as described by Tang et al. (1). These sites could be clinically active or non-active. However, the most commonly involved sites assessed by ultrasound are clinically active (lines 66-69).***

- **Point 2.** In addition, it is not appropriate to define oligoarthritis, polyarthritis, dactylitis and enthesitis as symptoms as these are clinical presentations.

***According to the reviewer’s suggestion, the word “symptoms” has been replaced by “clinical presentations” (lines 69-74) (2,3).***

- **Point 3.** In order to give greater strength to their work, the Authors should remember that psoriatic arthritis has peculiar effects on the bone and that the prevalence of osteoporosis in this condition is currently subject of wide debate. In fact, in psoriatic arthritis, close to erosive manifestations we also observe productive manifestations ranging from periostitis to ankylosis. The greatest complication of osteoporosis in clinical practice are fragility fractures which represent a significant clinical aspect. In this regard I would recall the work of Del Puente A and co-workers published in the Journal of Rheumatology in 2015 ( J. Rheumatol Suppl.2015 Nov;93:36-39).

***This issue related to fragility fractures occurrence in PsA patients has been addressed in lines 74-78. We also discussed the issue of osteoporosis prevalence in PsA (4,5) (lines 74-78).***

- **Point 4.** Finally, the patient studied must be accurately described.

What kind of psoriatic arthritis did he have: axial, peripheral or an overlap subset (axial and peripheral)?

Given the young age of the patient, it is necessary to specify how long psoriasis and arthritis had started.

What was the extent of psoriasis (calculated by PASI)?

***Additional information about the PsA patient has been added. We mentioned that the patient was affected by axial and peripheral psoriatic arthritis with the first symptoms reported 6 months before the first medical appointment. Moreover, cutaneous vulgaris psoriasis was detected in elbow and knee with a Psoriasis Area Severity Index (PASI) = 1.8 (lines 108-112).***

**References**

1.	Tang Y, Cheng S, Yang Y, Xiang X, Wang L, Zhang L, et al. Ultrasound assessment in psoriatic arthritis (PsA) and psoriasis vulgaris (non-PsA): which sites are most commonly involved and what features are more important in PsA? Quant Imaging Med Surg. gennaio 2020;10(1):86–95.

2.	Coates LC, Helliwell PS. Psoriatic arthritis: state of the art review. Clin Med. febbraio 2017;17(1):65–70.

3.	Ritchlin CT, Colbert RA, Gladman DD. Psoriatic Arthritis. Longo DL, curatore. N Engl J Med. 9 marzo 2017;376(10):957–70.

4.	Del Puente A, Esposito A, Costa L, Benigno C, Del Puente A, Foglia F, et al. Fragility Fractures in Patients with Psoriatic Arthritis. The Journal of Rheumatology Supplement. 1 novembre 2015;93(0):36–9.

5.	Attia EAS, Khafagy A, Abdel-Raheem S, Fathi S, Saad AA. Assessment of osteoporosis in psoriasis with and without arthritis: correlation with disease severity: Assessment of osteoporosis in psoriasis. International Journal of Dermatology. gennaio 2011;50(1):30–5.

***Detailed answers to Reviewer 2***

The study title is focused on the bone microarchitecture changes in psoriatic arthritic patient but the data is focused on the male population. This may be additional limitation because there are differences in the architecture and metabolism in male and female bones. Images were acquired in the arthritic psoriasis regions. Usually there are other high bone turnover regions that can be used to monitor bone quality changes. My proposition is to consider future study in typical regions to confirm the thesis that changes are not only connected with the disease involved regions. Because of the UHF MR pixel size additional studies with microCT should be considered. There is no data of the psoriasis intensity on screening.

***According to the reviewer’s suggestion, we added as a limitation the fact that the study was conducted in male only and that due to architecture and metabolism differences between male and female, future studies in female and in other bone regions could be of interest (lines 361-365). We mentioned that cutaneous vulgaris psoriasis was present in elbow and knee with a Psoriasis Area Severity Index (PASI) = 1.8 (lines 108-112).***
